# Biochar-Supported TiO_2_-Based Nanocomposites for the Photocatalytic Degradation of Sulfamethoxazole in Water—A Review

**DOI:** 10.3390/toxics9110313

**Published:** 2021-11-18

**Authors:** Subhash Chandra, Pravin Jagdale, Isha Medha, Ashwani Kumar Tiwari, Mattia Bartoli, Antonio De Nino, Fabrizio Olivito

**Affiliations:** 1 Department of Civil Engineering, Vignan’s Institute of Information Technology (A), Duvvada, Visakhapatnam 530049, India; subhash2k6@gmail.com; 2 Center for Sustainable Future Technologies, Italian Institute of Technology, Via Livorno 60, 10144 Torino, Italy; pravin.jagdale@iit.it (P.J.); mattia.bartoli@iit.it (M.B.); 3 Department of Mining Engineering, Indian Institute of Technology Kharagpur, Kharagpur 721302, India; 4 School of Environmental Sciences, Jawaharlal Nehru University, New Delhi 110067, India; ashwaniktiwari@mail.jnu.ac.in; 5 Department of Chemistry and Chemical Technologies, University of Calabria, Via P. Bucci, 87036 Rende, Italy; antonio.denino@unical.it

**Keywords:** sulfamethoxazole, photocatalysis, biochar, titanium oxide and antibiotic

## Abstract

Sulfamethoxazole (SMX) is a frequently used antibiotic for the treatment of urinary tract, respiratory, and intestinal infections and as a supplement in livestock or fishery farming to boost production. The release of SMX into the environment can lead to the development of antibiotic resistance among the microbial community, which can lead to frequent clinical infections. SMX removal from water is usually done through advanced treatment processes, such as adsorption, photocatalytic oxidation, and biodegradation. Among them, the advanced oxidation process using TiO_2_ and its composites is being widely used. TiO_2_ is a widely used photocatalyst; however, it has certain limitations, such as low visible light response and quick recombination of e^−^/h^+^ pairs. Integrating the biochar with TiO_2_ nanoparticles can overcome such limitations. The biochar-supported TiO_2_ composites showed a significant increase in the photocatalytic activities in the UV-visible range, which resulted in a substantial increase in the degradation of SMX in water. The present review has critically reviewed the methods of biochar TiO_2_ composite synthesis, the effect of biochar integration with the TiO_2_ on its physicochemical properties, and the chemical pathways through which the biochar/TiO_2_ composite degrades the SMX in water or aqueous solution. The degradation of SMX using photocatalysis can be considered a useful model, and the research studies presented in this review will allow extending this area of research on other types of similar pharmaceuticals or pollutants in general in the future.

## 1. Introduction

Water is an important resource for the living of animals, aquatic life, and human beings. During past decades, a continuous increase in the worldwide population and industrial activities have rapidly increased the water demand among the communities. Therefore, it is very much necessary to fulfil the water demand among the communities while maintaining the ecological balance [1]. However, due to the rapid industrialization and change in living style, the water quality in the natural resources such as rivers, lakes, and groundwater has substantially deteriorated due to the release of the pollutants [2,3]. In recent years, the production of antibiotics from pharmaceutical industries and their consumption by humans and animals has significantly increased due to the frequent endemic and pandemic episodes such as COVID-19, Ebola, and Swine flu [4,5]. The high consumption of antibiotics by animals and human beings led to their excessive release into the environment [6]. The antibiotics remain poorly metabolized and can enter into the environment through various pathways such as human waste effluent, pharmaceutical waste, agricultural run-off, wastewater treatment plant effluent, and effluent waste from livestock farming [7,8,9]. Antibiotics are usually categorized as contaminants of emerging concern (CECs) due to their persistence in the environment. The availability of antibiotics in the environment either in altered or in metabolized forms enables the bacteria to develop a resistance against them, which is a point of major concern for public health due to the possibility of increasing the occurrence of related clinical infections [10]. Among the reported antibiotics, sulfamethoxazole (SMX) is of prime concern due to its widespread global uses, its ability to solubilize in water, and binding to the soil or organic matter through the cation exchange process [10]. The SMX, which is the most commonly used sulfonamide, is frequently prescribed to treat a wide variety of bacterial infections such as ear, urine tract, respiratory, and intestinal infections in human beings and other infections in aquaculture and livestock farming to boost production [11]. It is considered a persistent organic pollutant because its metabolites are stable compounds with certain toxicity [12]. Various studies reported the presence of SMX in the rivers, lakes, groundwater, and effluents of the water treatment plants having concentrations varying from 40–370 ng/L [13,14,15]. Conversely, the concentrations of SMX in the effluents from various wastewater treatment plants, pharmaceutical industries, and landfills are range from ng/L to several μg/L [16,17,18]. Therefore, the removal and degradation of this compound in the natural water system and effluent discharge is important to reduce its adverse effect on human beings, animals, and the ecosystem.

Several techniques of SMX removal from water have been mooted in the earlier studies consisting of adsorption using graphene and carbon nanomaterials, biodegradation, degradation using sonocatalysis, degradation under gamma radiation using Fe/C nanoparticles-based metal-organic framework (MOF), and oxidation using UV/H_2_O_2_ treatment [19,20,21,22,23]. All the reported methods have certain shortcomings; for example, the sonocatalysis process requires high energy to produce high-energy soundwaves that limit its scaling-up process, and the UV/H_2_O_2_ oxidation process is associated with the problem of generating toxic transformation byproducts [24,25]. In this context, the photocatalytic oxidation method has gained much attention for the degradation of SMX in water due to its low operational cost and high efficiency.

Various types of photocatalytic materials, such as TiO_2_, ZnO and their carbon nanotube composites, were reportedly used for the photocatalytic degradation of SMX in water [26,27,28]. Among them, the TiO_2_ nanoparticle was frequently used for the photocatalytic degradation of antibiotics under UV light exposure or in a combination of TiO_2_ and H_2_O_2_ under UV exposure due to the availability of e^−^/h^+^ pairs, low band energy gap (3.20–3.35 eV), and high photocatalytic activities in the ultraviolet (UV) region [29]. However, the use of TiO_2_ as the photocatalytic material for the degradation of SMX in water is very limited due to problems associated with the low adsorption capacity, high agglomeration, and quick recombination of e^−^/h^+^ within the narrow wavelength range (200–400 nm) [30]. Many studies came up with the solution to dope the TiO_2_ nanoparticles with certain metals and carbon nanotubes to increase its photocatalytic sensitivity in the UV-visible range and reduce the band energy gap [31,32,33]. However, doping of the TiO_2_ with the metals is associated with the problem of the release of the metals into the environment, which can have a toxic effect on the ecosystem [34]. Conversely, the doping of TiO_2_ with CNTs is associated with the high cost of production, chances of increased recombination of e^−^/h^+^ pairs due to oxygen vacancies in bulk, and very limited long-term efficiency [30]. Hence, the integration of TiO_2_ nanoparticles with biomass waste-derived carbon material called “biochar” has been mooted as a novel solution to counter the above-reported problems in many of the earlier studies [34,35].

Biochar is a porous carbon material derived through the pyrolysis of biomass waste in the temperature range of 300–800 °C [36]. The integration of biochar as a supportive material for TiO_2_ nanoparticles was reportedly done to increase the adsorption of SMX onto the micro- and mesopores of biochar, reduce the band energy gap, and reduce the chances of the recombination of e^−^/h^+^ pairs due to the high semi-conductor activity of the material [37]. The motivation for writing a review on the photocatalytic degradation of SMX in water using the biochar-TiO_2_ composite material lies in the fact that, till now, various reviews have been published on the photocatalytic degradation of dyes, organic pollutants, and other antibiotics. However, not a single review is available particularly focusing on the speciation of SMX in water and its photocatalytic degradation using the biochar-supported TiO_2_ composite. The objectives of this paper are to review the following:

(i) Synthesis methods of biochar-supported TiO_2_ nanoparticles;
(ii) The effect of the integration of biochar with TiO_2_ particles in terms of changes in the physicochemical properties and increase in the photocatalytic response under UV-visible light;
(iii) Delineate the photochemistry of SMX in water and its major sources into the environment;
(iv) Delineate the chemical pathways and mechanisms involved during the photocatalytic degradation of SMX in water using the biochar-supported TiO_2_ nanoparticles.

## 2. Recent Degradation Techniques of the Common Antibiotics

Previous study results indicated that the application of TiO_2_-biochar composite showed up to 90% removal of SMX from the aqueous solution via photocatalysis under UV-visible radiation [38]. Another study showed that more than 80% of SMX was removed from the aqueous solution having high chemical oxygen demand via photocatalysis using the TiO_2_/biochar composite material [39]. Avramiotis and co-workers [40] developed a procedure to oxidize sulfamethoxazole through the activation of the persulfate by a pre-synthesized rice husk biochar, and they focused the study on the crucial activity of either electron transfer/singlet oxygen control and surface-bound radicals. In another recent work [41], in which SMX was indicated as a dangerous and recalcitrant pollutant, this antibiotic was degraded by the surface coating of a ceramic membrane by a photo-Fenton catalyst, and the degradation reached up to 90%. In a similar work carried out by Liu and co-workers [42], sulfonamide antibiotics were degraded efficiently using a combination of persulfate and UV irradiation. Vignati et al. [43] proved that ZnO can accelerate the photocatalytic degradation of Spiramycin in urban wastewater, and they carried out an in-depth study of the kinetics of the degradation and toxicity of the metabolites. In another work [44], the photocatalytic degradation of tetracycline hydrochloride under visible light irradiation using a newly synthesized photocatalyst MVO_4_/g-C_3_N_4_ (M = La, Ga) prepared by a hydrothermal method was investigated.

## 3. Sulfamethoxazole (SMX) in Water

### 3.1. Sources of Sulfamethoxazole in Water and Their Environmental Impacts

**Figure 1:**
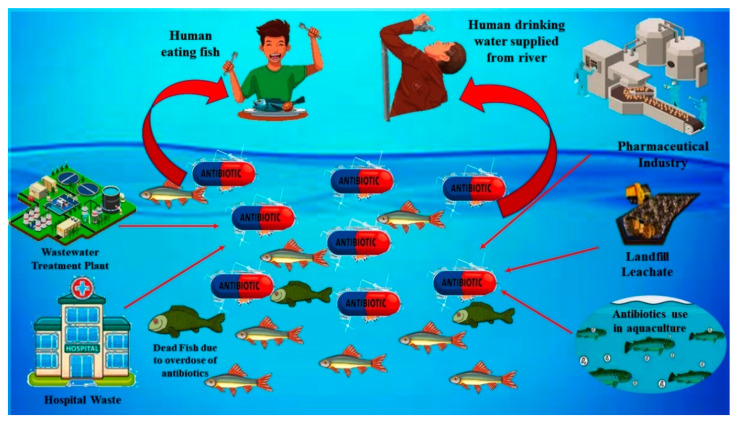
Graphical illustration of various point and non-point sources of SMX into the rivers and water resources.

There are various point and non-point sources of SMX in rivers and other water resources. The major sources of SMX (antibiotic) into water are illustrated graphically in Figure 1. The primary sources of this molecule into the water resources can be classified into five different groups: (i) effluent wastes from hospitals, (ii) effluent waste from pharmaceutical industries, (iii) effluent waste from the water treatment plant, (iv) effluent waste from aquaculture system and livestock farming, and (v) effluent and leachates from sanitary landfills [45]. The concentration of SMX in the river stream and water reportedly varies from ng/L to mg/L [45]. The SMX from the aquaculture system and livestock farming mostly come into the river stream through the disposal of wastewater generated from the farms directly into the river. The concentration of SMX from aquaculture systems (fish and shrimp) and livestock farms (pig and poultry) reportedly varies from 10 μg/L to 7 mg/L as reported in the water samples collected from shrimp ponds and canals nearby the livestock farming in Vietnam [46,47]. Untreated sewage is another source of SMX into the water as, during the heavy rainfall, untreated sewage, due to having limited hydraulic capacity, finds its way into the freshwater line. The concentration of SMX from the sewage discharge reportedly varies from ng/L up to 60 μg/L, as indicated by the data of the analysis of water samples from the sewage line discharge and freshwater streams within its nearby areas [48]. Apart from that, the presence of SMX in the effluent of wastewater treatment plants was also observed in the water samples, as reported in studies conducted in different countries [49,50]. The concentration of this compound in the effluent of sewage treatment plants reportedly varies from 226 to 3000 ng/L as per the data obtained from different studies conducted in China, Vietnam, and the Philippines [47,51,52]. Leachate from landfills is another major source of SMX discharge into the surface and groundwater. However, very little information and understanding of the mechanism is available regarding the fate and transport of this compound from the landfill sites. The concentration of SMX in the leachates reportedly varies from 6.4 to 8488 ng/L based on the data gathered from the studies conducted in the landfill sites of China and Singapore [53,54]. Moreover, the effluent waste from the pharmaceutical industries and hospital wastes are categorized as one of the direct sources of SMX and other antibiotics into the water. Previous studies carried out in the cities of China and Vietnam indicated the presence of SMX and other antibiotics in wastewater generated from hospitals and pharmaceutical industries [55,56]. The typical concentration of SMX in the wastewater generated from the pharmaceutical industry and hospitals reportedly varied from 320–2910 ng/L based on the data of the studies done earlier in China and Vietnam [55,57].

### 3.2. Photochemistry of Sulfamethoxazole in Water

**Figure 2:**
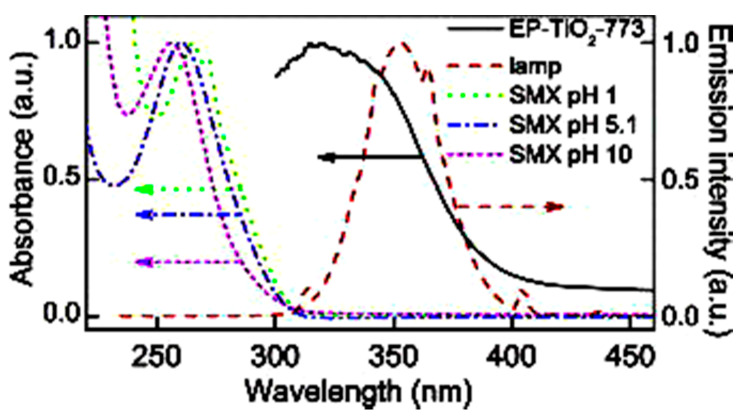
Variations in the wavelength absorbance by SMX with the change in the pH of the aqueous solution (reproduced from [64], Copyright Year 2015, Journal of Hazardous Material @ Elsevier with the permission order number: 5114720328428) (EP-TiO_2_: Expanded perlite coated TiO_2_ particles).

**Figure 3:**
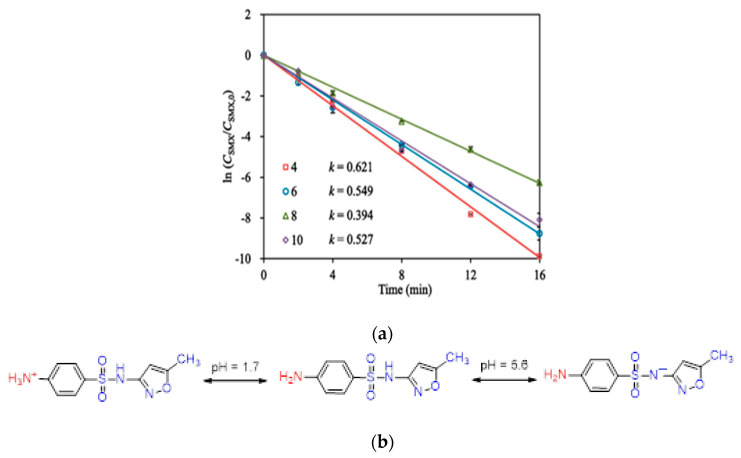
(**a**) Variations in the photocatalytic degradation of SMX concerning change in the pH of the aqueous solution (Reproduced from [22] (with the permission order number: 5114291052991); (**b**) charge variation in the structure of SMX with respect to the change in pH from acidic to alkaline in the aqueous solution (adapted from [64], Copyright Year 2015, Journal of Hazardous Material @ Elsevier with the permission order number: 5114720328428).

**Figure 4:**
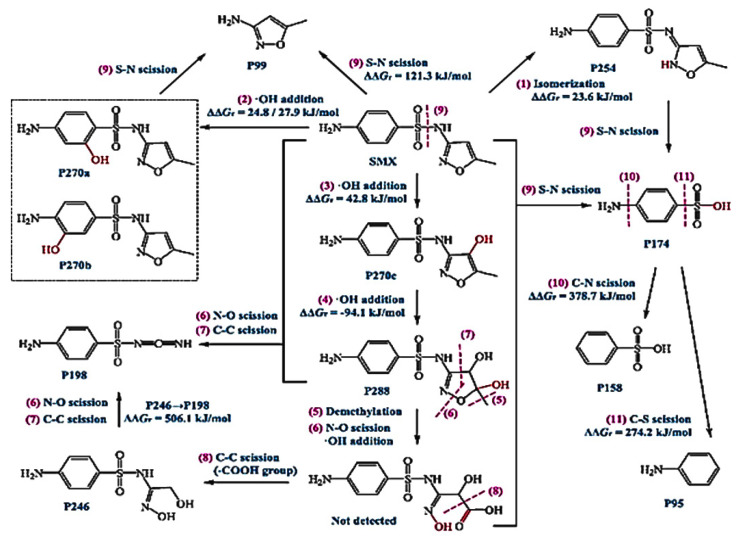
Mechanism of photolytic oxidation/reduction of SMX and the formation of various transformative products (TPs) during its photolysis (reproduced from [28] Copyright Year 2019, Chemical Engineering Journal @Elsevier with permission License number: 5114291052991© Elsevier).

Sulfamethoxazole (SMX) and tetracycline are widely used antibiotics, which are mostly used for the treatment of human beings and animals [58]. These antibiotics are usually detected in wastewater, surface water, and groundwater in the detection range of ng L^−1^ to μg L^−1^ [59,60]. Specifically, sulfamethoxazole (SMX), which comes under the chemical class of sulfonamide compounds, is an antimicrobial drug with broad-spectrum activity against gram-positive and negative bacteria [61]. The pharmaceutical waste, of which the antibiotic is a part, is usually hydrolyzed in water. The understanding of the degree and behavior of hydrolysis of antibiotics is of prime concern to understand their stability and non-biodegradability in the environment [62]. Sulfamethoxazole possesses good chemical stability in the environment, which allows it to resist metabolic processes and natural degradation [63]. The SMX usually absorbs the incident radiation light in the wavelength range of 250 to 300 nm, which also varies with respect to the change in the surface charge density on the SMX as the pH of the aqueous solution varies from acidic to alkaline [64]. The absorbance range reportedly shifted towards a higher wavelength side (up to 300 nm) as the pH of the aqueous solution was reduced to 1 from 10 (Figure 2) [64]. The pH of the solution plays a key role in the photocatalytic decomposition of SMX in water, as the SMX degradation rate reportedly was decreased with the increase in the pH value of the aqueous solution, as shown in Figure 3a [28]. The SMX mostly exists in the anionic form if pH > 5.6, and in the neutral form if pH lies between 1.85–5.6 (Figure 3b), as the pK_a1_ and pK_a2_ values of SMX was reported to be about 1.85 and 5.60, respectively [28,64]. The photolytic degradation of SMX is very difficult as it cannot be completely mineralized during oxidation [28]. Previous studies elucidated the photocatalytic oxidation mechanism of SMX and the formation of intermediatory transformation products (TPs) [28,64]. The detailed mechanism of SMX photodegradation in the presence of reactive oxygen species (ROS), i.e., •OH radical is shown in Figure 4. It can be observed from the figure that SMX formed its isomerization intermediatory product and is denoted by P 254. Subsequently, with the addition of •OH radical, the SMX is degraded to an intermediatory product denoted by 270 c. Thereafter, the addition of •֗OH radical to the transformation product (TP) defined as 270 c led to the demethylation process, which resulted in the formation of a new TP denoted by P 288. Subsequently, the breakage of the N–O bond and the removal of the carboxylic group from P 288 lead to the formation of another TP denoted by P 246. Additionally, the release of N–O and C–C bonds from P 288 formed another intermediatory product or TP denoted by P 198. The cleavage of the S–N bond from the SMX structure was reportedly occurred after reacting with an •OH free radical following the binding of N to an H atom, which leads to the formation of another TP called 3-amino 5-methylisoxazole denoted as P 99 [64]. The isomerized product of SMX is denoted by P 254, which, after reacting with •OH free radical, leads to the scission of S–N bond from P 254 that leads to the formation of a TP denoted by P 174. The P 174 on further oxidation with ROS leads to the formation of two new TPs denoted as P 158 and P 95, respectively, following the loss of C–N and C–S bonds.

## 4. Synthesis of Biochar-Supported TiO_2_ Nanocomposites

Titanium oxide (TiO_2_) is a conventionally used a very common type of photocatalytic material reported in many of the earlier studies for the photocatalytic oxidation/reduction of the pollutants in the aqueous solution due to having a very stable chemical structure and non-toxicity [65]. Despite having photocatalytic properties and stable structure, nowadays, TiO_2_ nanoparticles are not commonly used for the photocatalytic oxidation of pollutants in water. The reason behind its limited use for photocatalytic actions can be linked to its high bandgap (3.20 eV) and very narrow light absorbance in the ultraviolet-visible (UV-visible) range (200–400) [65,66]. Hence, TiO_2_ nanoparticles were reportedly chemically integrated with various materials, such as Zn nanoparticles, graphene, carbon nanotubes (CNT), activated carbon, and biochar, to reduce the band energy gap and increase the UV-visible absorbance range [67,68,69,70]. Among the above-reported supporting materials to synthesize the TiO_2_ composite, biochar has gained much attention due to its sustainability and low cost compared to the other reported materials. Moreover, the biochar-based nanoparticles reportedly have varieties of tunable functional groups, high chemical and thermal stability, and high electrical conductivity that, when integrated with the TiO_2_ nanoparticles, can reduce the bandgap and quick recombination of e^−^/h^+^ pairs during photocatalysis [38]. The biochar-supported TiO_2_ composite can be developed using various physical, chemical, and thermal methods whose details are comprehensively covered in the subsequent sub-sections.

### 4.1. Sol–Gel Method

**Figure 5:**
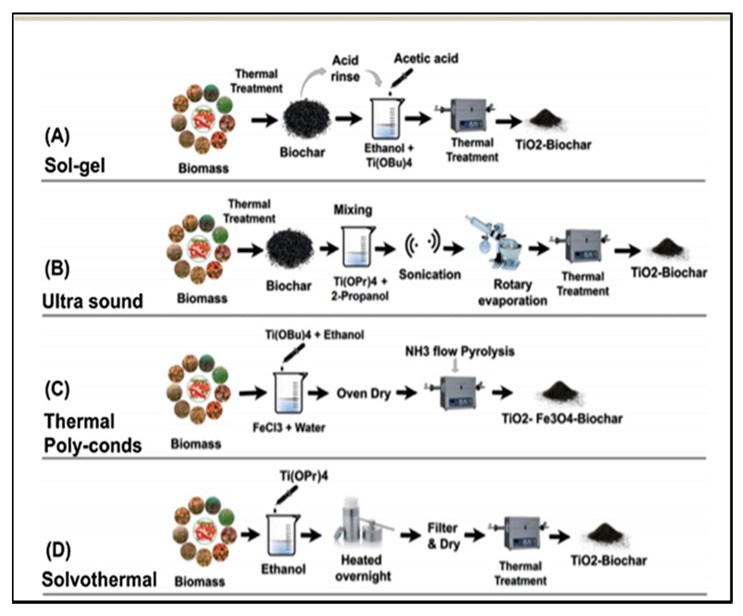
Illustration of various methods to develop biochar-supported TiO_2_ nanocomposite material for photocatalysis: (**A**) Sol-gel method, (**B**) Ultra sound method, (**C**) Thermal Polycondensation method, and (**D**) Solvothermal method for the synthesis of biochar supported TiO_2_ nanocomposite (reproduced from [73] Copyright year 2018, RSC Advances with permission under Creative Commons Attribution 3.0 Unported Licence).

Sol–gel is the most widely used method reportedly used for the synthesis of biochar-supported TiO_2_ nanocomposites (Figure 5A) [71,72,73]. In this method, the biomass is converted to biochar through the pyrolysis process [36], and then, the produced biochar is chemically treated with some weak acids, such as acetic or acrylic acid, to increase the surface oxides and reduce its pH [74,75]. Subsequently, the acid-treated biochar is then mixed with the slurry containing TiO_2_ nanoparticles in ethanol. The entire slurry mixture is then filtered through 0.22 μm filter paper to separate solid from liquid fraction. The solid fraction of the slurry is then calcinated in the temperature range of 500–700 °C to produce a highly stable biochar-supported TiO_2_ nanocomposite [76,77]. In the calcination process, the calcination temperature plays an important role, as heating the biochar TiO_2_ mixture above 700 °C reportedly changes the TiO_2_ crystal structure from anatase to rutile form [77].

### 4.2. Ultrasound Method

The ultrasound method is similar to the sol-gel method with the addition of an extra process of ultrasonication. The sonication of the pristine biochar with TiO_2_ in the organic aqueous solution is done to promote the impregnation of TiO_2_ nanoparticles into the pore of the porous biochar structure, which, upon calcination, reportedly provides a surface for TiO_2_ crystal growth [78]. The detailed procedure of the synthesis of biochar-supported TiO_2_ nanocomposite using the ultrasound method is illustrated in Figure 5B. Briefly, the biomass is converted to biochar through the pyrolysis process. The produced biochar after the crushing and sieving process is mixed with the Titanium isopropoxide and 2-propanol solution in a certain weight-by-volume ratio, and then, the mixture is subjected to the ultrasonication process for around 1–2 h. The ultrasonication of biochar and Titanium isopropoxide is done to break the biochar particles to nanoscale and to produce cracks on its surface, in which, subsequently, the TiO_2_ nanoparticles are reportedly embedded [76]. The TiO_2_-embedded biochar nanoparticles are then calcinated in the temperature range of 500–700° to start the nucleation of TiO_2_ crystals within the cracks present in the biochar particles to produce a biochar-supported TiO_2_ nanocomposite material.

### 4.3. Thermal Polycondensation Method

The thermal polycondensation process is also known as the single-step heating process to produce biochar-supported TiO_2_ nanoparticles, as illustrated in Figure 5C [79,80]. In this method, the biomass is initially mixed with some precursor such as melamine or polysaccharide agar to increase its thermal response during the calcination process. After that, the resultant biochar is mixed in a diluted ethanol solution containing titanium butyrate. The resulting solution mixture is oven-dried and then subjected to the calcination process under an ammonia gas environment to decrease the band energy gap and improve the visible light response of the biochar-supported TiO_2_ nanocomposite during the photocatalysis process [76,80].

### 4.4. Solvothermal Method

Solvothermal is the combination of hydrothermal treatment and dry heating process that facilitates the growth of TiO_2_ crystals onto the biochar’s surface, as shown in Figure 5D. In this method, the biomass is dispersed into an ethanol solution containing titanium isopropoxide in different (*w*/*w*) ratios, such as 1:1 or 1:2, and then, the entire mixture containing biochar particles and titanium isopropoxide is subjected to a hydrothermal process in the temperature range of 160–175 °C for a duration of 12–14 h [71,72]. Subsequently, the solids are separated from the mixture solution through the filtration process. The collected solid particles are washed several times with distilled water and then subjected to a calcination process in the temperature range of 600–700 °C for 6–8 h. The resultant is usually termed as biochar-supported TiO_2_ nanocomposite particles [73].

## 5. Effect of Biochar Addition on the Chemical and Structural Characteristics of TiO_2_ Nanoparticles

**Figure 6:**
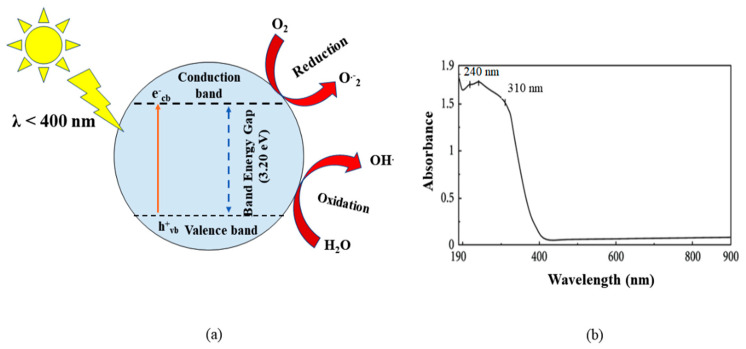
(**a**) Mechanism of generation of charged radicals from TiO_2_ upon illumination within wavelength less than 400 nm; (**b**) UV-visible absorbance by TiO_2_ particles within the wavelength range of 200–800 nm (reproduced from [77] Copyright 2012, International Journal of Photoenergy with permission under creative commons attributions 4.0 License).

Titanium dioxide is the most commonly used photocatalytic material reportedly being used for the photocatalytic degradation of pollutants in water under an advanced oxidation process (AOP). The pollutants are degraded by the charged radicals generated from TiO_2_ when illuminated under light with a wavelength less than 400 nm, as shown in Figure 6a. However, the use of TiO_2_ as a photocatalytic material for the oxidation/reduction of pollutants has certain limitations, such as a narrow photocatalytic activity region (λ < 400 nm), i.e., the very limited photocatalytic activity of the materials within the wavelength of the visible range (401–700 nm) (Figure 6b), quick recombination of e^−^/h^+^ pairs due to having large energy bandgap (~3.20 eV), very poor affinity towards organic pollutants, such as antibiotics, and the problem of agglomeration [74]. Hence, to overcome the limitations, the combination of the TiO_2_ nanoparticles with a sustainable porous carbon material called “biochar” to produce biochar-supported TiO_2_ nanocomposite for enhanced photocatalytic activities was mooted in the earlier studies [75,76].

**Figure 7:**
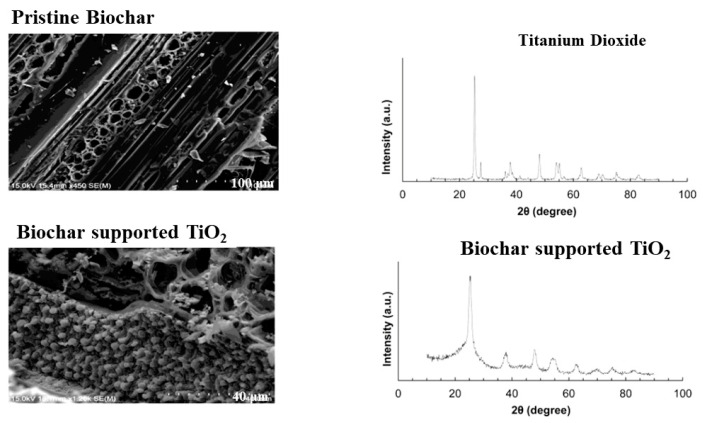
SEM and XRD pattern of the pristine biochar and biochar-supported TiO_2_ nanoparticle (reproduced with permission from [38] Copyright Year 2016, Journal of Environmental Management © Elsevier Pvt Ltd. License number 5122010796759).

**Figure 8:**
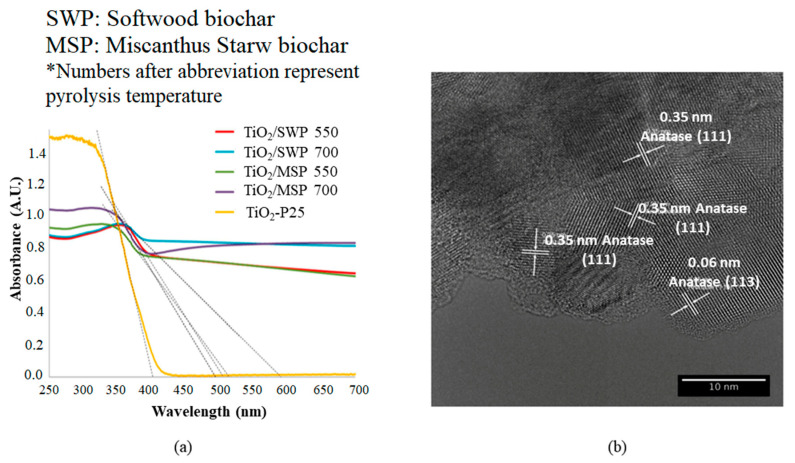
(**a**) UV-Visible absorbance of TiO_2_ and biochar-supported TiO_2_ (BSTPs) nanocomposites; (**b**) High-resolution transmission electron microscope image (HRTEM) of BSTPs (reproduced with permission from reference [78] copyright © 2021, American Chemical Society).

The rationale behind the integration of biochar particles with TiO_2_ nanoparticles can be linked to the fact that the presence of biochar with TiO_2_ nanoparticles reportedly increased the adsorption of pollutants on the composite’s surface, thereby increasing the photocatalytic degradation rates. Moreover, the biochar addition to the TiO_2_ nanoparticles reportedly increased the available electrons in the conduction band to generate charged oxygen and hydroxyl radicals through the reduction and oxidation process, respectively, due to the availability of free electrons on the biochar’s surface [70]. The major changes observed after the integration of biochar with the TiO_2_ nanoparticles were the change in the morphological structure, UV-visible absorbance range, and mineralogical structures, as shown in Figure 7 and Figure 8. It can be observed from Figure 7 that the biochar has a porous carbon structure that, after integration with TiO_2_ particles, provided a nucleation site during the calcination process within the pores for the crystallization of TiO_2_ nanoparticles within the biochar’s pore structure that can be seen in the form of granules on the biochar’s surface [38]. Additionally, the diffraction peaks at 2θ angles of 25.3, 37.8, 48.1, and 54.1 in the biochar-supported TiO_2_ reportedly indicate the presence of anatase TiO_2_ within the biochar structure, which confirms the successful integration of TiO_2_ in the biochar structure [38]. Further, the peaks at the 2θ angles of 62.5 and 70° are reportedly due to the presence of carbonates and CaO in the biochar’s structure [64]. The UV-visible absorbance (Figure 8a) showed that the TiO_2_ did not show any absorbance in the visible range (λ = 400–700 nm) but showed an absorbance of 1.4 (A.U) within the UV range (λ = 200–400 nm). However, the UV-visible absorbance of biochar-supported TiO_2_ showed a significant increase in the absorbance within both UV and visible light range unlike only in the UV range, as shown by the TiO_2_ nanoparticles. Apart from that, by increasing the pyrolysis temperature of the biochar from 550 to 700 °C, the UV-visible range light absorbance in the biochar-supported TiO_2_ nanocomposite was significantly increased due to an increase in the available free electrons in the biochar structure [78,79]. The high-resolution transmission electron microscopy (HRTEM) image of the biochar-supported TiO_2_ nanocomposite reportedly showed the presence of anatase TiO_2_ on the carpet-like biochar’s surface [78]. The HRTEM image (Figure 8b) confirmed the proper integration of TiO_2_ particles within the biochar’s structure. Thus, it can be observed that the integration of TiO_2_ nanoparticles with the biochar’s structure significantly changed the chemical characteristics of the resulting biochar-supported TiO_2_ nanocomposite. This makes it an efficient photocatalytic material for the photocatalytic degradation of the pollutants in the aqueous solution by increasing the light absorbance range, decreasing the e^−^/h^+^ recombination, reducing the band energy gap, and increasing the surface interaction of the pollutants with the nanocomposite surface, thereby increasing its photocatalytic activity.

## 6. Application of Biochar-Supported TiO_2_ Nanoparticles for the Photocatalytic Degradation of Sulfamethoxazole

**Figure 9:**
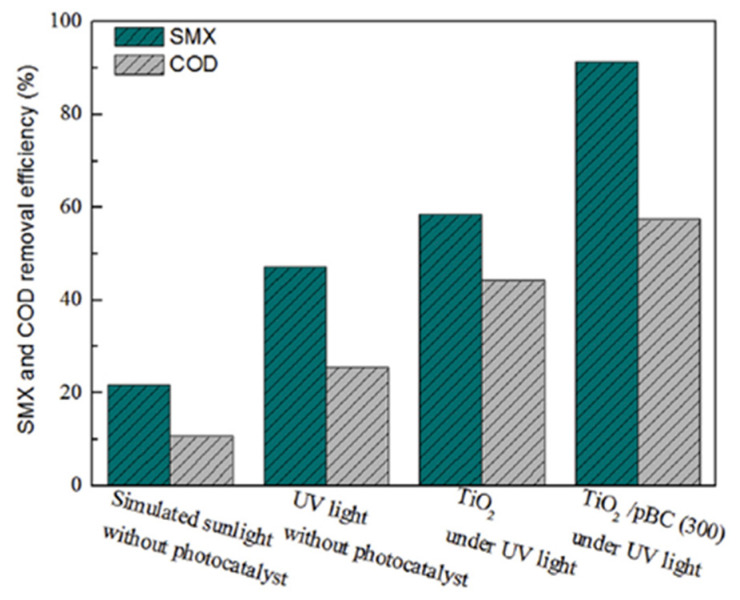
Photochemical degradation of SMX and COD under sunlight and UV light using TiO_2_/pBC composite (Initial SMX concentration = 10 mg/L, Photocatalyst dose = 0.2 g) (Reproduced from [88] Copyright Year 2017, Chemosphere with the permission License number: 5141190415626).

The removal of SMX using various techniques such as ozonation, reverse osmosis, membrane filtration, adsorption, biological treatments, and advanced oxidation has been mooted in the previous studies [61,80,81,82,83,84,85]. Among them, the advanced oxidation process is an emerging technique for the removal of the antibiotic from water due to the low cost of material synthesis, simple operation, and high efficiency of removal [86]. The removal efficiency of the wastewater containing SMX using the biochar-supported TiO_2_ and its comparison with respect to the only TiO_2_ under UV light source, without any photocatalyst under UV light, and without any catalyst under simulated sunlight is shown in Figure 9. It can be evinced from the figure that SMX degradation under simulated sunlight and UV light source without any addition of photocatalyst was very appreciable (20–50%) [87]. However, the removal efficiency of SMX with the addition of TiO_2_ as a photocatalytic material under UV light illumination was increased to up to 60%. Furthermore, this removal efficiency was substantially increased to more than 90% when the solvent was mixed with biochar-supported TiO_2_ composite photocatalytic material, indicating the higher photocatalytic activity of the biochar-TiO_2_ composite compared to the only TiO_2_ particles. Moreover, it can also be observed that the SMX was photo catalytically removed in the presence of COD in the solvent, whose removal percentage was also reportedly increased from 42 to 60% with the addition of biochar-TiO_2_ compared to the singularly applied TiO_2_ particles. Thus, the above result suggested that the removal efficiency of SMX was not compromised due to the presence of other organic and inorganic pollutants under photocatalytic degradation.

In Table 1, the data related to the photocatalytic removal of SMX from the water using the biochar-TiO_2_ composite, TiO_2_-CNT composite, and other TiO_2_ nanocomposites are given. It can be observed from the table that among all the listed TiO_2_ composites, the maximum removal efficiency of SMX from the water was shown by the biochar-TiO_2_ composite (91.27%) produced using the sol–gel method under the UV range (200–400 nm), followed by ZnO-TiO_2_ biochar composite (81.21%) and the multiwalled carbon nanotube (MWCNT)/TiO_2_ composite (90%). The reduced graphene oxide (RGO)/TiO_2_ showed a removal efficiency of 77.27% for the SMX removal from water or aqueous solution. Apart from the above-mentioned TiO_2_ composites, all the other forms of TiO_2_ composites, such as Cu-TiO_2_, clay-TiO_2_, and TiO_2_-borosilicate composites, reportedly showed a relatively lower SMX removal efficiency in water or aqueous solution (70–80%, Table 1). Moreover, it can also be observed that the photocatalytic removal efficiency of SMX using biochar/TiO_2_ was significantly (*p* < 0.05) decreased from 91.27 to 40.58% with the increase in the pH of the aqueous solution from 4 to 10.77. The decrease in the photocatalytic removal efficiency of SMX using the biochar/TiO_2_ composite with the increase in the pH of the aqueous solution can be linked to the pKa values of the SMX and biochar/TiO_2_ composite. The SMX reportedly has two pKa values (pKa = 1.7 and pKa = 5.6), which suggests that the SMX remained in the cationic form if the pH of the solution is less than 1.7 and in the anionic form if the pH > 5.6 units [89]. Additionally, the pKa value of the biochar/TiO_2_ composite was 6.1, indicating that the surface of biochar/TiO_2_ composite remained positively charged if the pH of the solution remained below 6.1; otherwise, it was negatively charged [90]. Hence, in the acidic pH condition (pH = 4), the biochar/TiO_2_ surface remained positively charged, which might have electrostatically attracted the negatively charged structure of SMX on its surface for adsorption. After adsorption of the SMX on the biochar/TiO_2_ surface, the SMX is reportedly degraded by the •OH radical produced via photocatalysis of the biochar/TiO_2_ composite. The integration of biochar as a supportive material with the TiO_2_ reportedly helps the photocatalytic degradation of organic pollutants in two ways: firstly, by providing the adsorption sites on its porous surface due to the availability of pores and high specific surface area, and secondly, having the ability to prevent the recombination of e^−^ and h^+^ pair due to having an abundance of π-electrons and low energy bandgap that eventually promotes the generation of •OH from the catalyst [91]. The detailed mechanism of SMX photocatalytic degradation in the aqueous solution is illustrated in Figure 9. The biochar/TiO_2_ photocatalytic composite reportedly photogenerates a h^+^ and e^−^ pair upon incidence with high photon energy that is sufficient enough to excite the h^+^ and e^−^ pair from the valance to conduction band. The photogenerated h^+^ is freely available on the surface of TiO_2_, which, upon reaction with H_2_O, reportedly produces •OH radical [92]. Similarly, the freely available e^−^ in the conduction band of TiO_2_ reportedly reacts with O_2_ to generate O_2_^−^ [93,94]. The detailed reactions to produce •OH and O_2_^−^ are given in Equations (1)–(6) [88]. Both of the radicals, i.e., OH and O_2_^−^, reportedly have a very strong ability to oxidize the SMX and its intermediates to some inorganic forms [88]. The chemical reactions depicting the photocatalytic generation of •OH and O_2_^−^ are given in Equations (1)–(6) as follows [77]:

TiO_2_/BC + hν → h^+^ + e^−^ (1)

h^+^ + H_2_O → •OHּ + H^+^ (2)

OH^−^ + H^+^ → •OH (3)

e^−^ + O_2_ → O_2_^−^ (4)

O_2_^−^ + H_2_O → •OH_2_ + O_2_^−^ (5)

•OH_2_ + H_2_O + e^−^ → H_2_O_2_ + OH^−^ (6)

**Figure 10:**
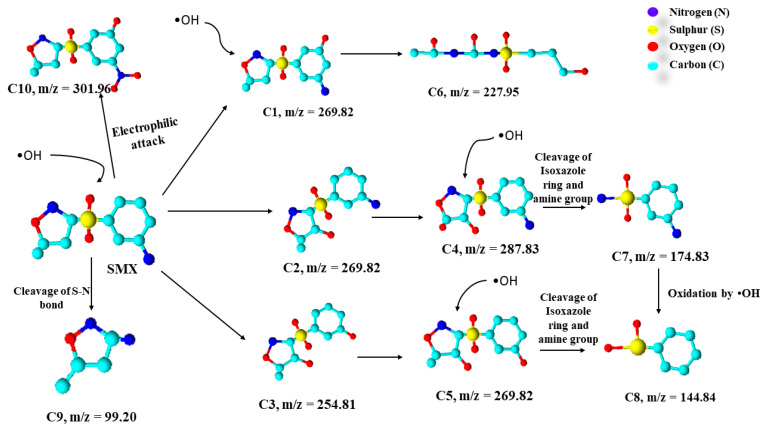
Photochemical degradation mechanism of SMX in water (adapted from [87,88] Copyright Year 2016 and 2017, Journal of Hazardous Materials and Chemosphere with permission License numbers: 5191250118976 and 5191390107382).

The generated •OH and O_2_^−^ radicals oxidize the SMX in the aqueous solution through three different pathways: hydroxylation, the opening of the isoxazole ring, and cleavage of the S–N bond [87]. Several intermediates produced during the photochemical degradation of SMX in water through •OH and O_2_^−^ radicals have been identified using HPLC chromatogram in a previous study [88]. During the hydroxylation process, the hydroxyl radical •OH reportedly attacks the benzene ring, isoxazole ring, and the amine group in the SMX. The detailed mechanism of the photochemical degradation of SMX in water or aqueous solution is illustrated in Figure 10.

From the figure, it can be observed that the SMX, upon oxidation by the hydroxyl radical (•OH), deteriorates in three different ways. First is the hydroxylation process, in which a hydroxyl group is attached to the carbon ring, the isoxazole ring, and relaces the amine group in the carbon ring, leading to the formation of three intermediates C1, C2, and C3 with *m*/*z* values of 269.81 and 254.81, respectively. The intermediate C1 upon further oxidation by the •OH leads to the formation of a stable product denoted by C5 with an *m*/*z* value of 227.95. Moreover, the further oxidation of C2 and C3 intermediates leads to the attachment of the oxygen group in the isoxazole ring, resulting in the formation of another two intermediates, namely C4 and C5 with *m*/*z* values of 287.33 and 269.82, respectively. These intermediates (C4 and C5) upon further oxidation by •OH radical reportedly cause the cleavage of the isoxazole ring from the carbon ring attached through the link of the sulphate group, resulting in the formation of stable end products denoted as C7 and C8 with m/z values of 174.83 and 144.84, respectively [88]. Besides this, there are two more ways through which the SMX oxidizes to the stable end products. Firstly, through the cleavage of S–N bond in the SMX due to oxidation by •OH radical that leads to the formation of end-product denoted by C9 with an m/z value of 99.20.

Besides this, a separate comparison has been discussed with respect to the synthesis techniques used for the synthesis of AC, biochar, reduced graphene oxide (rGO), and metal-doped TiO_2_ based photocatalysts and the effect of the various synthesis methods on the surface area and removal efficiency of various antibiotics (Table 2). It can be observed from the table that various synthesis methods, such as sol–gel, solvothermal, ultrasound method, thermal treatment, and hydrothermal methods, were used to synthesize AC, biochar, and metal-doped TiO_2_ photocatalysts. Among the reported methods, it was found that the AC-based TiO_2_ composite developed through the sol–gel method showed the highest removal efficiency of antibiotics (Tetracycline), followed by the rGO/TiO_2_ composite developed through the hydrothermal method. Subsequently, the BC/Zn/TiO_2_ and BiOBr/BC composites developed through the solvothermal method have shown high antibiotic removal efficiency of 96.8 and 92%, respectively. Conversely, the least organic pollutant removal was shown by the BC-TiO_2_ composite developed through the ultrasound-promoted wet impregnation method. Moreover, an interesting result was observed that the photocatalytic degradation of antibiotics was not much influenced by the surface area of the photocatalyst; rather, it depends on the synthesis method, the doping material being utilized for the development of the photocatalyst, as well as the pore volume of the photocatalyst. As far as the synthesis cost is concerned concerning the efficiency of the photocatalysts, it is beyond the scope of the present work. However, based on the technical methods and efficiency of the processes, it can be said that the sol–gel and hydrothermal are the most efficient and suitable methods to synthesize nanoscale photocatalysts having very high efficiency for the degradation of antibiotics in water.

## 7. Conclusions and Future Prospective

In the current perspective, the following conclusions can be deduced from the review:

(1) The use of a biochar-supported TiO_2_ composite for the photocatalytic degradation of antibiotics is an attractive method due to its high efficiency and low cost of operation compared to the existing treatment processes.
(2) The integration of biochar with the TiO_2_ nanoparticles increased photocatalytic degradation of SMX by increasing its photocatalytic response in the UV-visible range (200–700 nm) and the interaction of SMX with the TiO_2_ through the adsorption onto the biochar/TiO_2_ composite interface.
(3) The biochar-supported TiO_2_ composite can remove up to more than 95% of SMX in the aqueous solution within the UV range and up to 75% efficiency in the visible range.
(4) Unlike the doped photocatalyst, the biochar-supported TiO_2_ nanoparticles degrade the sulfamethoxazole both by adsorption and photocatalysis process and could also be used for the photocatalytic degradation of other antibiotics.
(5) The •OH free radical is the prime key component that degrades the sulfamethoxazole through the oxidation or reduction process.

In the future, more studies focusing on the application of biochar-supported TiO_2_ composites for the photocatalytic degradation of antibiotics in the natural water stream under sunlight irradiation are required. Moreover, future studies should pay more attention to enhancing the adsorption capacity of biochar by doping it with certain metal or non-metal elements to increase the adsorption of anionic antibiotics onto the biochar-TiO_2_ surface to increase the photocatalytic degradation. Additionally, more studies are required focusing on the photocatalytic degradation of SMX using a biochar/TiO_2_ composite in real water under open sunlight as very limited studies are available delineating the photocatalytic degradation mechanism of SMX using the biochar/TiO_2_ composite.

## Figures and Tables

**Table 1:** Photocatalytic degradation of Sulfamethoxazole (SMX) at different experimental conditions using biochar-supported TiO_2_ nanocomposite and other hybrid TiO_2_ composites.

S. No.	Type of Photocatalyst	Synthesis Method	pH	Light Irradiation Range	SMX Removal (%)	References
1	UV light without photocatalyst	-	4	UV range (200–400 nm)	47.24	[88]
2	TiO_2_	-	4	UV range (200–400 nm)	58.47	
3	TiO_2_/Biochar	Sol–gel	4	UV range (200–400 nm)	91.27	
4	TiO_2_/Biochar	Sol–gel	5.95	UV range (200–400 nm)	82.24	
5	TiO_2_/Biochar	Sol–gel	8.53	UV range (200–400 nm)	65.17	
6	TiO_2_/Biochar	Sol–gel	10.77	UV range (200–400 nm)	40.58	
7	RGOT/SA (Reduced graphene oxide TiO_2_/Sodium alginate)		-	UV range (200–400 nm)	77.6	[95]
8	MCNT/TiO_2_ (Multiwalled carbon nano tube/TiO_2_)	Acid catalysed Sol–gel	-	UV range (200–400 nm)	90	[96]
9	TiO_2_/Biochar	Sol–gel	-	UV range (200–400 nm)	91	[38]
10	ZnO-TiO_2_/Biochar	Modified Sol–gel	3.95	UV irradiation (λ < 410 nm)	78.34	[39]
11	ZnO-TiO_2_/Biochar		5.03		81.21	
12	ZnO-TiO_2_/Biochar		6.92		75.48	
13	ZnO-TiO_2_/Biochar		8.95		71.10	
14	Clay-TiO_2_ composite	Sol–gel	-	UV irradiation	70.2	[97]
15	Cu-TiO_2_	Sol–gel	-	UV-visible (300–800 nm)	94%	[98]
16	TiO_2_-GAC-MPR (Activated Carbon-Membrane photobioreactor	Sol–gel	-	UV irradiation	83.60	[99]
17	TiO_2_-Borosilicate Glass	Solvothermal	-	UV irradiation	70	[100]
18	Bi_2_O_4_–TiO_2_	Hydrothermal	5.0	UV-Visible (190–1100 nm)	90	[101]

**Table 2:** Comparison of synthesis methods on the properties of carbon-based TiO_2_ nanocomposites and the removal efficiency of antibiotics.

S. No.	Composite Material	Synthesis Method Used	BET Surface Area (m^2^/g)	Pore Volume (cc/g)	Targeted Antibiotic	Mechanism of Removal	Removal Efficiency	References
1	AC/TiO_2_	Sol–gel	129	0.30	Tetracycline	Photocatalysis	~97%	[102]
2	BC/Zn/TiO_2_	Solvothermal	435	-	acetaminophen	Photocatalysis	92%	[103]
3	BC-BiOCl	One step hydrolysis	3,546	0.011	Tetracycline	Photocatalysis	60.3%	[104]
4	BC-TiO_2_	Ultrasound promoted wet impregnation	399	-	Phenol	Photocatalysis	64.1% (UV light) 55667733.6% (Visible light)	[78]
5	Magnetic BC/TiO_2_	Solvothermal	-	-	Sulfadiazine	Photocatalysis	~88%	[105]
6	TiO_2_/rGO	Hydrothermal	48.09	-	Sulfamethoxazole	Photocatalysis	~90%	[106]
7	rGO/TiO_2_/Na Alginate	Hydrothermal	-	-	Azithromycin	Photocatalysis	~99%	[81]
8	Bi/Bi_2_O_3_/BC	Thermal method	338.2	0.161	Estrone	Photocatalysis	~90%	[107]
9	BioBr/BC	Solvothermal	-	-	Ciprofloxacin	Photocatalysis	96.8%	[108]
10	CuWO_4_/BC	Hydrothermal	6.8104	-	Ciprofloxacin	Photocatalysis	97%	[109]

## Data Availability

Not applicable.
